# Protective Role of Whey Protein Isolate on MPP^+^-Induced Differentiation of SH-SY5Y Cells by Modulating the Nrf2 Antioxidant Pathway

**DOI:** 10.3390/molecules30102207

**Published:** 2025-05-18

**Authors:** Panlekha Rungruang, Morakot Sroyraya, Veerawat Sansri

**Affiliations:** 1 Center of Excellence for Dental Stem Cell Biology, Faculty of Dentistry, Chulalongkorn University, Bangkok 10330, Thailand; 2 Department of Anatomy, Faculty of Science, Mahidol University, Bangkok 10400, Thailand; 3 Department of Basic Medical Science, Faculty of Medicine Vajira Hospital, Navamindradhiraj University, Bangkok 10300, Thailand

**Keywords:** Parkinson’s disease, whey protein isolate, MPP^+^, Nrf2, antioxidant

## Abstract

The pathogenesis of Parkinson’s disease (PD) consists of the apoptosis of dopaminergic neurons in the substantia nigra pars compacta (SNpc) due to oxidative stress. The present study aimed to evaluate the potential antioxidant activity of whey protein isolate (WPI) in PD models, using neurotoxin-exposed SH-SY5Y cells differentiated into dopaminergic-like neurons. Our research shows that WPI’s high glutamic acid, aspartic acid, and leucine contribute to its antioxidant and neuroprotective effects, with glutamic acid crucial for glutathione synthesis. In vitro studies found that WPI, at concentrations of 5–1000 µg/mL, is non-toxic to differentiated SH-SY5Y cells. Notably, the lowest con-centration of WPI (5 µg/mL) significantly decreased intracellular reactive oxygen species (ROS) levels in these cells following a 24 h co-treatment with 1-methyl-4-phenylpyridinium (MPP^+^). The antioxidant effects of WPI were also confirmed by the increased expression of HO1 and GPx antioxidant enzymes, which are Nrf2 pathway target genes and were evaluated by real-time PCR. Furthermore, Nrf2 nuclear translocation in the differentiated SH-SY5Y cells was also increased when the cells were exposed to 5 µg/mL of WPI with MPP^+^. These results together suggest that WPI has antioxidant effects on dopaminergic-like neurons in a Parkinson’s disease model.

## 1. Introduction

Parkinson’s disease (PD) is a chronic neurodegenerative disease whose progression is characterized by the gradual loss of dopaminergic neurons in the substantia nigra pars compacta (SNpc) of the midbrain. The general motor symptoms include bradykinesia, muscle rigidity, and tremors [1]. The etiology of PD is complex and multifactorial, with oxidative stress playing a significant role in its progression [2]. The enhanced production of reactive oxygen species (ROS) increases the oxidative stress that damages lipids, proteins, and DNA leading to neuronal cell death [3].

The nuclear factor erythroid 2-related factor 2 (Nrf2) signaling pathway is the main cellular defense against oxidative stress [4]. Normally, Nrf2 is trapped in the cytoplasm by Kelch-like ECH-associated protein 1 (Keap1), which marks it for ubiquitination and subsequent degradation. However, under conditions of oxidative stress, Nrf2 is released from Keap1, translocates to the nucleus and activates the transcription of antioxidant response element (ARE)-driven genes. The genes included in this response are superoxide dismutase 1 (*SOD1*), glutathione S-transferase (*GST*), glutamate–cysteine ligase catalytic subunit (*GCLC*), glutathione peroxidase (*GPX*), heme oxygenase-1 (*HMOX1*), and NAD (P)H quinone dehydrogenase 1 (*NQO1*) [5,6,7,8].

Whey proteins are known to contain bioactive peptides with antioxidant, antihypertensive, and antimicrobial activities that are beneficial to the body when ingested [9,10]. Whey protein isolate (WPI) is a highly purified form of whey protein that is characterized by its high protein content, bioavailability, and functional properties, with further purification removing most of the lactose (<1%) and fat [11]. Due to its excellent solubility, digestibility, and rich amino acid profile, WPI has become increasingly popular in nutritional and therapeutic applications [12]. Beyond its nutritional value, WPI exhibits strong antioxidant potential [13]. Research has highlighted the presence of bioactive peptides in whey protein and its hydrolysates, which demonstrate significant antioxidant effects in both experimental models and clinical trials. Non-cellular antioxidant investigations, such as DPPH radical scavenging activity, ferric reducing capacity, free radical scavenging activity, and oxygen radical absorbance capacity, have confirmed the antioxidant properties of various hydrolyzed WPI derivatives derived from bovine sources [14,15,16]. Several studies have demonstrated that bioactive peptides released from whey protein or its hydrolysates can exert antioxidative effects by scavenging ROS, enhancing glutathione synthesis, and modulating cellular redox signaling pathways. For example, such peptides have been reported to prevent oxidative stress in cystic fibrosis mouse models [17] and to activate immune responses in mouse spleen lymphocytes [18]. WPI has been investigated in a variety of systems, including chronic unpredictable stress mouse models [19], Saos-2 human osteoblast-like cells [20], and HUVECs [21]. More recently, WPI supplementation has been evaluated in older adults and found to alter serum levels of IL-12p70 and IL-13 [22]. In patients with type 2 diabetes, WPI was reported to improve inflammation and oxidative stress markers [23]. Additionally, mice fed a high-fat diet supplemented with WPI exhibited reduced expression of pro-inflammatory markers (MCP-1, TNF-α, and CD68) in the ileum and epididymal white adipose tissue compared to mice fed a control casein-based diet [24]. Nevertheless, the precise mechanisms through which WPI influences oxidative stress and the Nrf2 signaling pathway in PD cell models have not yet been fully elucidated.

SH-SY5Y cells, a human neuroblastoma cell line with dopaminergic characteristics, are widely used in PD research due to their ability to model dopaminergic neuron-like behavior upon differentiation. The differentiation of SH-SY5Y cells using RA and TPA results in the elevated expression of tyrosine hydroxylase (TH), dopamine-β-hydroxylase, and the dopamine transporter (DAT) [25,26]. Additionally, genes involved in dopaminergic function, including those regulating dopamine synthesis, release, reuptake, degradation, and signaling, such as dopamine receptor D2 (DRD2) and vesicular monoamine transporter 1 (SLC18A1), show higher expression in differentiated SH-SY5Y cells compared to undifferentiated cells [27]. MPP^+^, the active metabolite of MPTP, induces selective mitochondrial dysfunction and oxidative stress by entering dopaminergic neurons via DAT and disrupting mitochondrial complex I, making it a well-established in vitro model for studying PD-related neurotoxicity [28].

The present study was carried out to establish the potential antioxidant properties of WPI on MPP^+^-induced oxidative stress in the differentiating SH-SY5Y cells, which are widely used in the in vitro model of PD. As such, we intend to determine the potential neuroprotective mechanisms of WPI by determining intracellular ROS levels, Nrf2 nuclear translocation, and the expression of key antioxidant genes (*SOD1*, *GST*, *GCLC*, *GPX*, *HMOX1*, and *NQO1*). These findings provide preliminary evidence supporting the potential of WPI to mitigate oxidative stress in PD-related cellular models.

## 2. Results

### 2.1. Total Amino Acids Contained in WPI

The total amino acid composition of the WPI sample is presented in Table 1. The analysis showed that glutamic acid was the most abundant amino acid with a concentration of 17,457.40 mg/100 g, followed by aspartic acid (10,920.43 mg/100 g), and leucine (10,441.27 mg/100 g). Lysine and threonine were also present in relatively high amounts, at 8342.23 and 7006.69 mg/100 g, respectively.

Moderate levels of isoleucine (5808.87 mg/100 g), proline (5747.21 mg/100 g), valine (5319.86 mg/100 g), alanine (4955.06 mg/100 g), and serine (4510.00 mg/100 g) were observed. Cystine and methionine, the sulfur-containing amino acids, were found at concentrations of 3323.42 and 2205.17 mg/100 g, respectively. Aromatic amino acids, phenylalanine (3206.23 mg/100 g) and tyrosine (2957.29 mg/100 g), were detected.

Lower quantities of arginine (2109.95 mg/100 g), histidine (1872.83 mg/100 g), glycine (1705.66 mg/100 g), tryptophan (1536.06 mg/100 g), and hydroxylysine (574.38 mg/100 g) were also determined. These findings indicate that WPI possesses a diverse amino acid profile that is abundant in both essential and non-essential amino acids, highlighting its nutritional value.

### 2.2. The Differentiation of SH-SY5Y Cells into Dopaminergic-like Neurons Using RA and TPA

**Figure 1:**
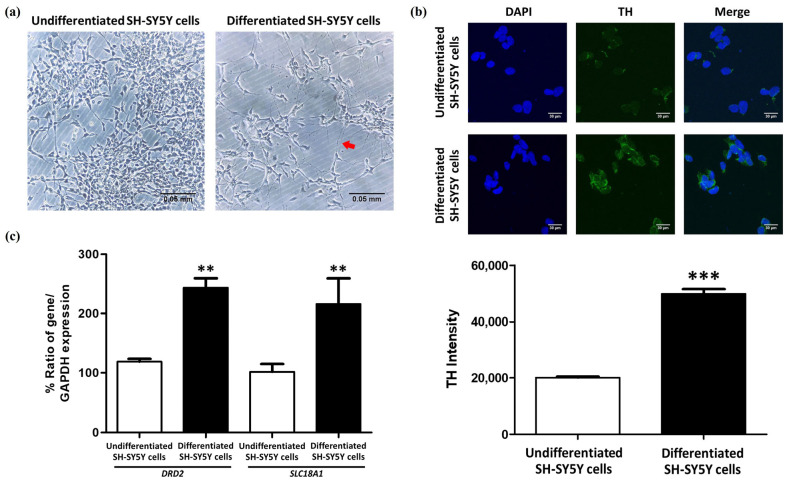
SH-SY5Y neurons were differentiated into dopaminergic-like neurons for use as PD models. (**a**) The morphology of undifferentiated SH-SY5Y cells and differentiated SH-SY5Y cells after incubation with and without 10 µM RA and 80 nM TPA for 6 days. The red arrow is the branching process connecting each cell after differentiation. (**b**) Immunofluorescent staining of TH in SH-SY5Y cells. TH is represented by green signals around the blue signal, which is the nucleus (scale bar = 30 μm). The intensity of TH in undifferentiated and differentiated SH-SY5Y cells is shown in the bar graph. (**c**) The relative expression of *DRD2* and *SLC18A1* genes in undifferentiated and differentiated SH-SY5Y cells. The data are presented as the mean ± SEM; n = 120 for TH staining, and n = 3 for RT-PCR. ** *p* ≤ 0.01 and *** *p* ≤ 0.001 indicate significant differences between groups.

After differentiating SH-SY5Y cells using 10 µM of RA for 3 days, followed by 80 nM of TPA for another 3 days [25,29,30], morphological analysis under an inverted microscope revealed elongated projections connecting the cells (Figure 1a). Tyrosine hydroxylase (TH) staining showed that the SH-SY5Y cells, which were induced to differentiate, had a significantly higher intensity of TH staining than the undifferentiated cells, with an increase of 87.93% (*p* < 0.001) (Figure 1b).

Gene expression analysis was performed to determine dopaminergic markers from dopamine transporter (DAT; encoded by *SLC18A1*) and dopamine D2 receptor (*DRD2*). Both *DRD2* and *SLC18A1* were found to be expressed at higher levels in the differentiated SH-SY5Y cells as compared to the undifferentiated cells [27]. In addition to TH staining, the quantification of these dopaminergic neuron markers was also performed, and there was a significant increase in the level of *DRD2* (*p* = 0.0016) and *SLC18A1* (*p* = 0.0057) in the differentiated SH-SY5Y cells by 124.549 and 124.266%, respectively (Figure 1c). These findings show that SH-SY5Y cells, upon differentiation, express features similar to dopaminergic neurons and, therefore, are a good model to study the pathogenesis of PD and for use in future experimental studies.

### 2.3. WPI Has Non-Toxic Effects on Differentiated SH-SY5Y Cells

**Figure 2:**
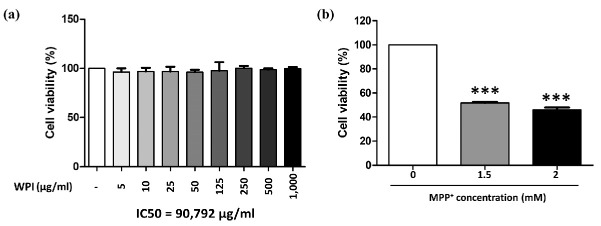
Differentiated SH-SY5Y cell viability after exposure to WPI (**a**) for 24 h or MPP^+^ (**b**) at various concentrations. The data are presented as the mean ± S.E.M.; n = 3. Statistical analysis revealed significant differences between the control group and test groups (*** *p* ≤ 0.001).

SH-SY5Y cells were incubated with WPI at concentrations ranging from 5 to 1000 µg/mL for 24 h. The results indicated that there was no significant difference in cell viability between the treated cells and the control group (0 µg/mL) after 24 h of treatment. The findings indicate that none of the tested concentrations of WPI caused toxicity to differentiated SH-SY5Y cells following 24 h of exposure (Figure 2a). Additionally, the IC50 value for WPI after 24 h of treatment was calculated to be 90,792 µg/mL, suggesting that only supraphysiological concentrations would adversely affect cell viability in this model. Moreover, the optimal concentration of MPP^+^ required to reduce differentiated SH-SY5Y cell viability by approximately 50%, thereby simulating dopaminergic neuronal loss observed in Parkinson’s disease, was determined. The results showed that exposure to 1.5 mM MPP^+^ reduced cell viability to 48.3% (*p* < 0.001), while 2 mM MPP^+^ caused a slightly higher reduction at 53.96% (*p* < 0.001), as shown in Figure 2b. Based on these findings, 1.5 mM MPP^+^ was selected as the optimal concentration for inducing Parkinsonian-like cytotoxicity in subsequent experiments.

### 2.4. WPI Reduces the Intracellular ROS in MPP^+^-Induced Differentiated SH-SY5Y Cells by Inducing Nrf2 Translocation into the Nucleus

**Figure 3:**
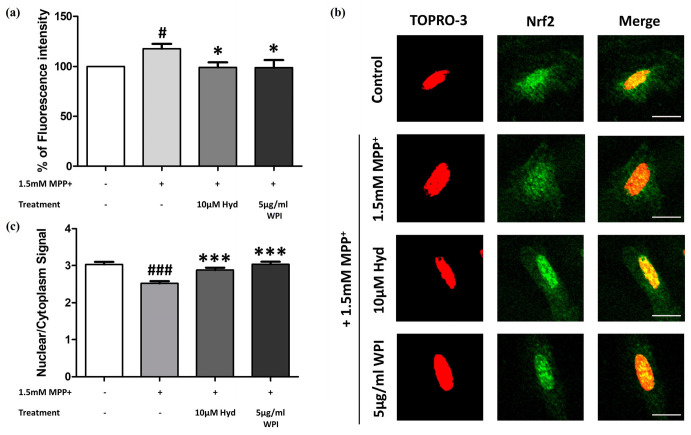
WPI decreased the production of intracellular ROS in MPP^+^-treated differentiated SH-SY5Y cells through the accumulation of Nrf2 in the nucleus. (**a**) The assessment of intracellular relative ROS levels in differentiated SH-SY5Y cells exposed to MPP^+^ and WPI, with Hyd used as a positive control. (**b**) The examination of Nrf2 nuclear translocation in differentiated SH-SY5Y after co-treatment with WPI and MPP^+^. Nrf2 nuclear translocation was confirmed by immunofluorescence staining using Nrf2 antibody (green) and TOPRO-3 dye (red) (scale bar = 100 µm). Merged images displaying a yellow color (resulting from the merging of green and red) within the nucleus indicate the successful nuclear translocation of Nrf2. (**c**) The quantification of the nuclear–cytoplasmic signal ratio. Data are presented as mean ± SEM; * *p* ≤ 0.05 and *** *p* ≤ 0.001 vs. MPP^+^-treated group, # *p* ≤ 0.05 and ### *p* ≤ 0.001 vs. control group.

Subsequently, we aimed to establish whether WPI is capable of reducing intracellular ROS in differentiated SH-SY5Y cells that were stimulated by MPP^+^ by increasing the activity of an antioxidant pathway. Differentiated SH-SY5Y cells were co-treated with 1.5 mM of MPP^+^ and 5 µg/mL of WPI for 24 h. The results showed that WPI at 5 µg/mL (*p* = 0.024) significantly reduced fluorescence intensity by 18.714% compared to the MPP^+^-treated group (Figure 3a). The same effects were also observed with Hyd, which was used as a positive control and also reduced the level of ROS.

In addition, WPI may act as an antioxidant through the regulation of the Nrf2/ARE pathway, which controls the expression of antioxidant genes. To assess this, immunofluorescence staining was performed to evaluate Nrf2 nuclear translocation, a key event in the activation of detoxification and cytoprotective genes. As shown in Figure 3b,c, the nuclear green fluorescence signal of the cells treated with 1.5 mM of MPP^+^ was reduced compared to that of the cells treated with 5 µg/mL of WPI and MPP^+^. This indicated that Nrf2 nuclear translocation in differentiated SH-SY5Y cells co-treated with MPP^+^ and 5 µg/mL of WPI was statistically increased (*p* < 0.001) and was 1.206 times higher than that of the cells treated with MPP^+^ only. The nuclear/cytoplasmic green signal was similar to that of the cells treated with 10 µM of Hyd (positive control).

### 2.5. WPI Increases the Expression of Antioxidants Through the Nrf2 Signaling Pathway

**Figure 4:**
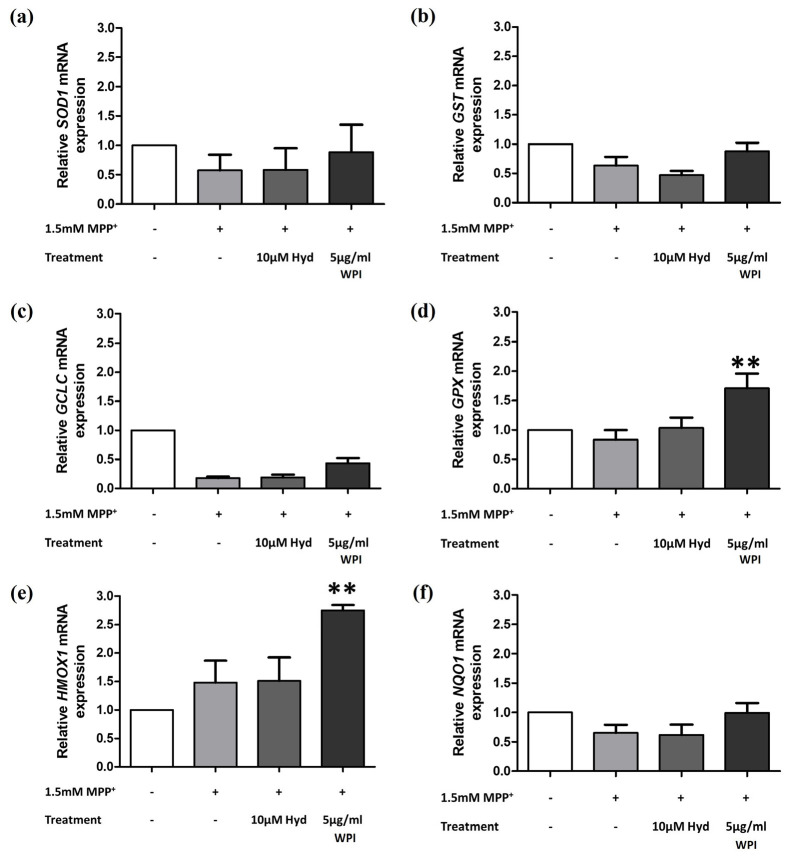
The expression of antioxidant genes in differentiated SH-SY5Y cells was assessed following co-treatment with MPP^+^ and WPI. (**a**–**f**) The mRNA levels of *SOD1*, *GST*, *GCLC*, *GPX*, *HMOX1*, and *NQO1* were assessed using RT-qPCR. The data are presented as the mean ± SEM; n = 3. ** *p* ≤ 0.01 indicates significant differences compared to the MPP^+^ group.

Nrf2 nuclear translocation was first examined, followed by the quantitative assessment of downstream antioxidant gene expression, including *SOD1*, *GST*, *GCLC*, *GPX*, *HMOX1*, and *NQO1*, using RT-qPCR. The results of the gene expression analysis revealed that cells treated with 5 µg/mL of WPI and MPP^+^ had significantly increased levels of *GPX* and *HMOX1* compared to MPP^+^ alone, with increases of 2.047 and 1.855 times, respectively (*p* value of 0.004 and 0.008, respectively) (Figure 4d,e). The expression levels of *SOD1*, *GST*, *GCLC*, and *NQO1* showed increasing trends in the differentiated SH-SY5Y cells treated with 5 µg/mL of WPI and 1.5 mM of MPP^+^; however, these increases were not statistically significant (Figure 4a–c,f).

## 3. Discussion

In this study, the neuroprotective effects of whey protein isolate were investigated in MPP^+^-induced SH-SY5Y cells, which were differentiated into dopaminergic-like neurons. The SH-SY5Y human neuroblastoma cell line is widely used as a Parkinson’s disease model due to its dopaminergic-like morphology and expression of key markers, including TH, dopamine-β-hydroxylase, and the DAT [25,26]. The results of this investigation demonstrated that RA and TPA induction promoted morphological differentiation and increased TH expression in SH-SY5Y cells. Additionally, this treatment significantly upregulated the expression of DRD2 and SLC18A1, which are critical for dopamine neurotransmission. These findings align with previous studies [25,27,30,31,32], confirming that the differentiated SH-SY5Y cells are a suitable model for PD research. This study investigates the antioxidant effects of WPI using an MPP^+^-induced SH-SY5Y cell model of Parkinson’s-like neurotoxicity. While this model is widely used to study dopaminergic damage and oxidative stress, it has notable limitations. MPP^+^ is the active metabolite of MPTP, an exogenous neurotoxin that crosses the blood–brain barrier and is converted to MPP^+^, which selectively accumulates in dopaminergic neurons via the dopamine transporter (DAT). MPP^+^ disrupts mitochondrial complex I, leading to oxidative stress and neuronal cell death. However, this mechanism does not fully capture the slow, progressive, and multifactorial nature of idiopathic PD. It also lacks hallmark pathological features such as Lewy body formation and widespread non-dopaminergic involvement. Moreover, many compounds that showed efficacy in MPTP/MPP^+^ models have failed in clinical trials, raising concerns about their translational relevance [33]. Although MPP^+^ does not cause idiopathic PD, it remains a valuable tool for modeling specific cellular and molecular aspects of the disease, particularly mitochondrial dysfunction and oxidative stress, in experimental systems. It serves as a useful platform for screening potential neuroprotective agents such as WPI. Further studies using in vivo models are warranted to confirm these findings.

The amino acid analysis of WPI suggests that it can be used as a functional dietary supplement with potential neuroprotective and antioxidant effects. The five most common amino acids in WPI are glutamic acid, aspartic acid, leucine, lysine, and threonine, respectively. Glutamic acid is involved in the synthesis of the antioxidant reduced glutathione (GSH) and forms the GPX4-GSH antioxidant system with glutathione peroxidase 4 (GPX4), which is involved in the protection against oxidative stress and ferroptosis [34]. Furthermore, both glutamic acid and aspartic acid can enhance NRF2 activity within cells [35]. In addition, the carboxyl and amino groups in their side chains make these amino acids work as metal ion chelators and hydrogen donors [36,37]. Leucine, a hydrophobic amino acid, can prevent lipid peroxidation by serving as a proton acceptor [38]. Lysine enhances the activities of antioxidant enzymes by activating the NRF2 transcription factor and upregulating the expression of antioxidant enzyme genes, thus enhancing the capacity to decompose free radicals and to prevent oxidative stress [39].

The supplementation of threonine has been found to enhance muscle antioxidant capacity by regulating the Nrf2/Keap1 signaling pathway, reducing oxidative stress, and enhancing muscle growth in hybrid catfish [40]. WPI is rich in cystine, a precursor of glutathione (GSH), which is involved in redox balance and has independent antioxidant activity [41]. Cysteine is a radical scavenger, which protects cells from oxidative stress and helps in the regeneration of GSH [36,37]. Moreover, methionine, the other sulfur-containing amino acid, is involved in antioxidant defense through its nucleophilic sulfur moiety that can directly neutralize radicals [36,37]. However, it should be noted that the present study utilized intact, non-hydrolyzed WPI, which was directly applied to differentiated SH-SY5Y cells. As such, the immediate bioavailability of its amino acids and their direct cellular effects are likely dependent on intracellular uptake and subsequent proteolytic processing. Although we reported the native amino acid composition of WPI, the observed cellular effects are unlikely to be attributed solely to the intact protein. Rather, they may result from a combination of factors, including the partial hydrolysis of WPI by endogenous enzymes in the culture medium or by intracellular proteases following cellular internalization. These processes could lead to the generation of bioactive peptides or free amino acids that contribute to the antioxidant and neuroprotective effects observed. To better understand these mechanisms, further studies involving simulated gastrointestinal digestion or comprehensive peptidomic analysis are recommended to identify the specific bioactive components responsible for the observed outcomes.

To determine the neuroprotective and antioxidant potential of WPI, the co-treatment with MPP^+^ was performed to simulate the conditions of PD. Thus, the co-treatment was used to establish whether WPI is capable of decreasing the oxidative stress and cell damage induced by MPP^+^, a neurotoxin that blocks complex I of the electron transport chain in mitochondria and results in an overproduction of ROS, leading to the death of dopaminergic neurons [42]. Thus, the role of WPI in reducing intracellular ROS levels, upregulating antioxidant genes, and enhancing Nrf2 translocation into the nucleus was investigated. The outcomes of the present study thus reveal the possible protective mechanisms of WPI on dopaminergic-like neurons in a PD-like condition.

**Figure 5:**
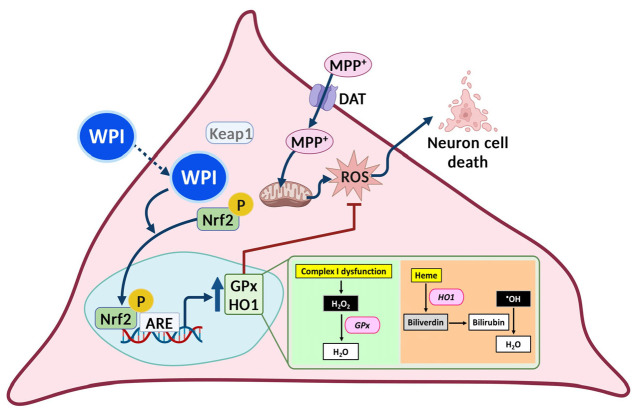
The schematic of the proposed mechanism illustrates that WPI, at a concentration of 5 µg/mL, exerts antioxidant properties on differentiated SH-SY5Y cells. WPI may be internalized by differentiated SH-SY5Y cells and exert bioactivity. WPI induces the translocation of Nrf2 into the nucleus, resulting in the enhanced expression of antioxidants such as *GPx* and *HO1*. Following this, these antioxidants decrease intracellular ROS, a precursor to cell death. Excessive intracellular ROS is induced by mitochondrial dysfunction caused by MPP^+^, which enters differentiated SH-SY5Y cells through the dopamine transporter (DAT). GPx converts H_2_O_2_ to water molecules. HO1 changes heme, which is a toxic molecule, to biliverdin, which is subsequently converted to bilirubin, which is an antioxidant molecule that can convert hydroxyl radicals (•OH) to water molecules. The figures were produced through the utilization of the BioRender.com platform.

From the results of this research, it was found that WPI did not exhibit cytotoxicity on differentiated SH-SY5Y cells after 24 h treatments. The high IC50 of WPI on differentiated SH-SY5Y cells indicates that, in case of an overdose, WPI may be safe for neuronal cells. This result is correlated to previous studies, in which whey protein isolate was studied for cytotoxicity on other cells, and the results revealed that whey protein isolate was not toxic to rat cerebellar granule neurons, NSC34 motor neuronal cells, or HT22 hippocampal cells and significantly protected neurons against diverse inducers of oxidative stress [43]. Moreover, WPI at a concentration of 5 µg/mL significantly reduced intracellular ROS levels in these cells when co-treated with MPP^+^. This reduction in ROS is consistent with the antioxidant properties of whey proteins, as outlined in a previous report [44], which highlights their ability to scavenge free radicals and mitigate oxidative stress. However, the antioxidant properties of WPI should be further evaluated using chemical-based antioxidant assays, such as the inhibition of liposome peroxidation, ferric reducing antioxidant power assay, metal-chelating activity assay, DPPH radical scavenging assay, reducing power assay, ABTS radical cation decolorization assay, oxygen radical absorbance capacity assay, or ferric reducing power assay. Results from these assays could provide additional evidence to support that WPI may act as a free radical scavenger. Reactive oxygen species (ROS) can be reduced by antioxidants produced via the Nrf2/ARE signaling pathway, which is a key cellular mechanism activated within the nucleus [45]. The effects of WPI on the Nrf2 translocation into the nucleus under the co-treatment condition with MPP^+^ was determined. The results revealed that WPI at 5 µg/mL highly facilitated the translocation of Nrf2 into the nucleus. The proposed mechanism is in accordance with the action of synthetic small molecules and natural compounds that alter the redox sensitive cysteine residues of Keap1 to control Nrf2 activation. Although peptide identification was not performed in the present study, WPI-derived components may interact with specific cysteine residues such as Cys151, Cys273, and Cys288 that are found in the regulatory domains of Keap1, altering its conformation and preventing the Keap1–Nrf2 interaction. These residues have been reported in the previous literature as critical sites for conformational changes in Keap1 that disrupt its interaction with Nrf2, thus allowing Nrf2 to translocate to the nucleus and activate antioxidant gene expression [46]. However, to strongly confirm the ability of WPI to interact with Keap1, the bioactive peptides contained in WPI should first be identified. This could be followed by in silico investigations, such as molecular docking, to assess potential peptide–protein interactions. Subsequently, in vitro assays capable of validating these interactions, such as ELISA, should be performed to confirm the predicted results. All of the present findings suggest that WPI may enhance antioxidant levels in differentiated SH-SY5Y cells under toxic conditions. The proposed mechanism is illustrated in Figure 5.

In the present study, WPI significantly enhanced the levels of antioxidant genes, *GPX* and *HMOX1*, in MPP^+^-co-exposed differentiated SH-SY5Y cells. GPx uses H_2_O_2_ to produce water, thus counteracting ROS generated by mitochondrial dysfunction [3]. Additionally, HO1 breaks down free heme, which reduces the pro-oxidant effects of heme and also prevents the formation of ROS through the Fenton reaction [47]. These actions help in the protection of dopaminergic neurons from oxidative damage and apoptosis in PD models. Furthermore, the enhanced Nrf2 translocation into the nucleus of our cells co-incubated with WPI and MPP^+^ was consistent with the previous studies that reported that the whey protein components are capable of stimulating Nrf2 expression and its nuclear accumulation in EA.hy 926 cells [48], HepG2 cells [49], and MDA-MB-231 cells [50]. The neuroprotective role of WPI in PD models is also supported by the earlier research on whey protein supplements. For instance, Tosukhowong et al. (2016) conducted a pilot study on PD patients, showing improvements in clinical symptoms, and they found a decrease in plasma homocysteine, which is an independent risk factor for cardiovascular disease, and an increase in plasma branched-chain amino acids and essential amino acids following whey protein supplementation [51]. Ross et al. (2012) reported that a cysteine-rich whey protein isolate acted as a neuroprotective agent in different neurodegenerative disease models by maintaining cellular glutathione levels [52]. These findings suggest that the antioxidant effect observed with WPI is part of a broader potential of whey proteins in managing neurodegenerative conditions.

For future studies, it is crucial to also determine the bioactive compounds in WPI that act to decrease intracellular ROS and activate Nrf2 nuclear translocation. Furthermore, it is important to determine whether WPI-derived compounds can cross the blood–brain barrier and act as neuroprotective agents in the central nervous system. A recent strategy involves blocking the direct interaction between Keap1 and Nrf2 through protein–protein interactions, thereby boosting Nrf2′s transcriptional activity by preventing its ubiquitination and subsequent degradation. This approach may be useful as a therapeutic for a number of medical conditions, including neurodegenerative diseases [53]. It has been proposed that direct inhibition by binding to the Kelch domain of Keap1 is of low risk and is physiologically acceptable for use in humans [54]. In addition, it is suggested that molecular docking should be carried out to predict the affinity of antioxidant peptides to the Nrf2 binding site on Keap1.

## 4. Materials and Methods

### 4.1. Chemicals and Reagents

In this study, we used the following supplies: D-MEM/Ham’s F-12 culture medium with L-Glutamine and phenol red, and D-MEM/Ham’s F-12 with L-Glutamine and Sodium Pyruvate (phenol red-free), both from FUJIFILM Wako Pure Chemical Corporation (Osaka, Japan). The fetal bovine serum (FBS) was obtained from Gibco (Life Technologies Korea, Seoul, Republic of Korea). The 0.25% Trypsin EDTA and 100× Penicillin-Streptomycin (Pen/Strep) solution were from ANH (New York, NY, USA). The substances used were 1-methyl-4-phenylpyridinium iodide (MPP^+^), retinoic acid (RA), 12-*O*-tetradecanoylphorbol-13-acetate (TPA), and 2′,7′-dichlorofluorescein diacetate (DCFH-DA), which were obtained from Sigma-Aldrich Corporation (St. Louis, MO, USA). Dimethyl Sulfoxide (DMSO) of analytical reagent (AR) grade was purchased from VWR Chemicals (Briare, France), and Thiazolyl blue tetrazolium bromide (MTT) was from Bio Basic (Amherst, NY, USA). Thermo Fisher Scientific (Waltham, MA, USA) supplied the Goat anti-rabbit IgG conjugated with Alexa Fluor^®^ 488, TO-PRO™-3 Iodide, TRIzol reagent (Thermo Fisher Scientific, Waltham, MA, USA), and Taq DNA Polymerase. The Anti-Tyrosine Hydroxylase antibody (ab6211) was purchased from Abcam (Cambridge, UK). The iScript Reverse Transcription Supermix and iTaq Universal SYBR Green Supermix were from Bio-Rad (Berkeley, CA, USA), and the NRF2 (D1Z9C) XP^®^ Rabbit mAb was from Cell Signaling Technology (Danvers, MA, USA). The water used was ultrapure DNase/RNase free, from Invitrogen Corporation (Waltham, MA, USA). The agarose powder was purchased from Vivantis (Shah Alam, Malaysia), and the Prime juice preloading fluorescent stain was obtained from Bio-Helix (New Taipei City, Taiwan). All other chemicals and reagents used were of analytical grade.

### 4.2. Total Amino Acid Analysis

The total amino acid composition of the samples was determined using two validated in-house methods, TE-CH-372 and TE-CH-373, as described in previous studies [55,56]. In brief, the samples were ground to pass through a 0.5 mm sieve. Hydroxyproline, glutamic acid, aspartic acid, leucine, lysine, threonine, isoleucine, proline, valine, alanine, serine, cystine, phenylalanine, tyrosine, methionine, arginine, histidine, glycine, and hydroxylysine were quantified using the in-house method TE-CH-372. These amino acids were analyzed after acid hydrolysis with 6 M of hydrochloric acid containing 1 g/L of phenol at 110 °C for 23 h. To prevent their degradation, the sulfur-containing amino acids (methionine and cysteine) were oxidized to methionine sulfone and cysteic acid using performic acid before hydrolysis. Tryptophan was analyzed by the TE-CH-373 in-house method, which needs alkaline hydrolysis using 4 M of sodium hydroxide at 110 °C for 20 h to preserve it.

Both methods’ hydrolysates were filtered through a 0.2 µm membrane filter and analyzed on an amino acid analyzer using a sulfonated polystyrene resin column. The amino acids were separated by ion-exchange chromatography, and then post-column derivatization was performed with ninhydrin. Detection was carried out at 570 nm for most amino acids and at 440 nm for proline. The quantitation of amino acids was based on the comparison of the sample peak areas with the peak areas of the calibration standards of individual amino acids. Norleucine was used as an internal standard to correct for variability during the analysis. Amino acid quantities were given in mg/100 g units.

### 4.3. SH-SY5Y Cell Culture and Differentiation

In this experiment, the SH-SY5Y cells were obtained from the American Type Culture Collection (Manassas, VA, USA). They were grown in D-MEM/Ham’s F-12 medium containing 10% (*v*/*v*) FBS and 1% (*v*/*v*) Pen/Strep solution. The cells were cultured in a humidified incubator at 37 °C with 5% CO_2_. Then, SH-SY5Y cells were seeded on culture plates and allowed to attach for 24 h. The medium was changed every 3 days, and the cells were allowed to grow to 80% confluency. To induce differentiation into dopaminergic-like neurons, the cells were first treated with 10 µM of RA for three days, followed by 80 nM of TPA for an additional three days in D-MEM/Ham’s F-12 medium containing 2% (*v*/*v*) FBS and 1% (*v*/*v*) Pen/Strep.

### 4.4. Reverse Transcription Polymerase Chain Reaction (RT-PCR)

Previous studies have used the genes that were significantly upregulated in the differentiated SH-SY5Y cells as markers for differentiation, such as dopamine receptor D2 (DRD2) and vesicular monoamine transporter 1 (SLC18A1) [27]. DRD2 and SLC18A1 were quantified by RT-PCR to see the biochemical differentiation of SH-SY5Y cells induced to become dopaminergic like neurons when compared to undifferentiated cells.

#### 4.4.1. RNA Isolation

For harvesting, undifferentiated and differentiated SH-SY5Y cells were collected from 6 well cell culture plates, which were seeded at a low density (4 × 10^5^ cells/well) and maintained under specific conditions. The TRIzol reagent was used to isolate the total RNA from the cells following the manufacturer’s protocol. First, the culture medium was removed, 330 µL of TRIzol reagent was applied to each well, and the solution was left to incubate for 5 min. The cells were then lysed and incubated for an additional 5 min. Subsequently, 200 µL of chloroform was added per 1 mL of TRIzol reagent, and the mixture was vigorously shaken by hand for 15 s, followed by a 5 min incubation at room temperature. The samples were then centrifuged at 12,000× *g* for 15 min at 4 °C. The supernatant from the top layer was then carefully transferred to a new tube, and 0.5 mL of isopropanol was added to it. The mixture was placed on the ice for 10 min. The mixture was then centrifuged at 12,000× *g* for 15 min at 4 °C. After the centrifugation, the supernatant was carefully removed using a micropipette, and the RNA pellet was washed with 1 mL of 75% ethanol. The sample was then centrifuged again at 10,000× *g*, 4 °C for 5 min. The supernatant was then removed using a micropipette, and the RNA pellet was then allowed to dry in the air at room temperature and was then resuspended in ultrapure distilled water. The RNA solution was kept at −80 °C until further processing.

#### 4.4.2. cDNA Synthesis

cDNA at a concentration of 0.125 µg/µL was prepared by converting 2.5 µg of RNA into cDNA. In brief, the reverse transcription master mix was chilled before using it. First, the PCR tube was filled with ultrapure distilled water, and then iScript Reverse Transcription Supermix, 4 µL per tube, was added. The template RNA was pipetted gently and then briefly centrifuged before adding it into each PCR tube. The reverse transcription master mix was inserted into the PCR machine after it was prepared. The thermal cycler conditions were primer annealing at 25 °C for 5 min, then reverse transcription at 46 °C for 20 min, and termination of the reverse transcription at 95 °C for 1 min. All cDNA samples were stored at −20 °C until further use.

#### 4.4.3. RT-PCR Performing

The expression levels of the dopaminergic marker genes *DRD2* and *SLC18A1* were ascertained using RT-PCR. In order to facilitate PCR amplification, a master mix was prepared, per reaction, which included 10X Taq buffer with (NH_4_)_2_SO_4_ at 2.5 µL, 2 mM dNTPs at 2.5 µL, 10 µM forward and reverse primers at 1 µL each, 25 mM MgCl_2_ at 3 µL, and ultrapure distilled DNase/RNase-free water at 11 µL, followed by the two last components, 0.125 µg/µL cDNA template at 4 µL and 5 U/µL Taq DNA polymerase at 0.25 µL. Finally, the PCR was conducted using the recommended thermal cycling conditions for amplification. RT-PCR conditions included initial denaturation at 95 °C for 3 min, denaturation of DNA at 95 °C for 30 s, annealing at 60 °C for 30 s, and extension at 72 °C for 1 min, subsequently repeating cycling from denaturation to extension 40 times. Finally, the final extension at 72 °C for 5 min was performed, and the DNA was held at 4 °C. The primer sequences used in this study were specifically designed to target the *DRD2*, *SLC18A1*, and *GAPDH* genes. For *DRD2*, the forward primer sequence (FW) was 5′-CCCTATGGCTTGAAGAGCCTG-3′, while the reverse primer sequence (RV) was 5′-AGTTGCCCTTTAGTGGAGCC-3′ [57]. The *SLC18A1* gene was amplified using the forward primer 5′-GGCTTTGCTATAGGCTATTCTGA-3′ and the reverse primer 5′-GGCATTTGGCAGCAAGACAA-3′ [58]. For the reference gene *GAPDH*, the forward primer was 5′-ACCTCAACTACATGGCTGAGAA-3′, and the reverse primer was 5′-CATGGCAACTGTGAGGAGGG-3′.

#### 4.4.4. Agarose Gel Electrophoresis

After RT-PCR was performed, the quantitative DNA of DRD2 and SLC18A1, as well as the housekeeping gene, GAPDH, were determined by performing agarose gel electrophoresis. Firstly, the 1.5% agarose gel was prepared in 0.5× TBE buffer and set in the gel tray. During the agarose gel setting, the DNA samples were prepared by mixing cDNA with Prime juice preloading fluorescent stain at a ratio of 5:1, respectively. Next, the DNA samples were loaded into a 1.5% agarose gel, and electrophoresis was run at 100 V and 400 mA for 40 min under 0.5× TBE. The DNA bands were then observed under the B-BOX™ Blue Light LED Epi-illuminator (SMOBIO, Paramount, CA, USA). The intensity of DNA bands was measured by utilizing the Image J software, version 1.52a (Java 1.8.0_112).

### 4.5. Preparation of WPI Solution

The WPI, HMS 90^®^ (IMMUNOTHAI Co., Ltd., Bangkok, Thailand), a pure whey protein powder derived from bovine milk, was diluted in 1× PBS and prepared into 5000 µg/µL (stock solution) by measuring the protein concentration by BCA assay.

### 4.6. Cell Viability Measurement

Cell viability was evaluated using the MTT assay after the differentiated SH-SY5Y cells were treated. Briefly, 100 µL of MTT solution at a concentration of 0.5 mg/mL was added to each well of a 96-well plate containing cells and incubated at 37 °C for 3 h. Afterward, AR-grade DMSO was added to each well to dissolve the formazan crystals. The plate was gently agitated in the dark for 10 min to ensure the complete dissolution of the formazan crystals, resulting in the formation of a purple solution. The OD measurements were taken using a Gemini XPS and EM Microplate Reader (Molecular Devices, San Jose, CA, USA). Absorbance was measured at 570 nm to quantify the formation of formazan, with a reference OD measurement at 690 nm to account for background noise.

### 4.7. Cytotoxicity Test of WPI in Differentiated-SH-SY5Y Cells

Using the MTT assay, cytotoxicity experiments were conducted to evaluate the potential toxicity of WPI on differentiated SH-SY5Y cells. Initially, SH-SY5Y cells were seeded in 96-well plates at a density of 8 × 10^3^ cells per well. The differentiated SH-SY5Y cells were subjected to a range of concentrations of WPI, including 5, 10, 25, 50, 125, 250, 500, and 1000 µg/mL, following the aforementioned differentiation stages. The WPI was diluted in D-MEM/Ham’s F-12, which was supplemented with 2% (*v*/*v*) FBS and 1% (*v*/*v*) Pen/Strep, and incubated for 24 h. The MTT assay was subsequently employed to evaluate cell viability. Furthermore, the GraphPad Prism 5.0 software was employed to determine the half-maximal inhibitory concentration (IC50) values of WPI at 24 h. Moreover, before performing the next experiments, the optimal concentration of MPP^+^ was collected. In brief, seeding of an incremental number of differentiated SH-SY5Y cells at 8000 cells per well, followed by exposure with MPP^+^ at concentrations of 1.5 and 2 mM for 24 h, was performed. The MPP^+^ concentration that induced cell death at around 50% was selected for future experiments.

### 4.8. Intracellular ROS Measurement

SH-SY5Y cells were plated in black 96-well plates with a transparent bottom (ANH, New York, NY, USA) at a cell density of 8 × 10^3^ cells/well. The cells were allowed to differentiate for 6 days. After the differentiation, the SH-SY5Y cells were treated and subjected to DCFH-DA to determine the intracellular ROS production. The DCFH-DA assay was performed as follows: The culture media was removed and cells were loaded with 10 µM DCFH-DA in 100 µL of D-MEM/Ham’s F-12 medium containing L-Glutamine and Sodium Pyruvate (without phenol red) without FBS and Pen/Strep. The cells were kept in the dark at 37 °C for 30 min. After the incubation, the solution was removed, and the cells were incubated with an additional 100 µL of D-MEM/Ham’s F-12 medium containing L-Glutamine and Sodium Pyruvate without FBS and Pen/Strep. The green fluorescence signal was quantified using a Microplate Reader (Spark 10M, Tecan Trading AG, Männedorf, Switzerland) at an excitation wavelength of 485 nm and an emission wavelength of 530 nm. Hydralazine (Hyd) was applied as a positive control for ROS production, Nrf2 translocation, and antioxidant gene expression since it is known to induce Nrf2 nuclear translocation and to increase the expression of antioxidant genes [59]. There were the following four experimental groups: a control group, an MPP^+^-treated group serving as the oxidative stress model, a Hyd co-treatment group with MPP^+^, and a WPI co-treatment group with MPP^+^.

### 4.9. Immunofluorescent Staining for Investigating TH and Nfr2 Translocation into the Nucleus

#### 4.9.1. TH Staining for Determining the Increase of Dopaminergic-like Neurons After Differentiation

SH-SY5Y cells (1.5 × 10^5^ cells/well) were seeded onto Poly-D-lysine-coated coverslips in 6-well plates (ANH, New York, NY, USA) and allowed to adhere for 24 h. Differentiation into dopaminergic-like neurons was induced, with undifferentiated SH-SY5Y cells prepared concurrently for comparison. Immunofluorescence staining was performed as follows: the cells were fixed with 4% paraformaldehyde, blocked with 5% normal goat serum in PBST, and incubated overnight with a primary antibody against TH (1:300). The next day, cells were incubated with a secondary antibody (Alexa Fluor^®^ 488, Thermo Fisher Scientific, Waltham, MA, USA, 1:1000) for 2 h in the dark. After washing with PBST, the cells were mounted with antifade reagent supplemented with DAPI. Images were acquired using an FV10i confocal laser scanning microscope (Olympus, Tokyo, Japan). The green fluorescence signals on cells were quantified, subtracting the background signal, using Image-J software, version 1.52a (Java 1.8.0_112).

#### 4.9.2. Nfr2 Translocation into the Nucleus Investigation

The immunofluorescence staining was performed following the previous study [60]. Briefly, cells were seeded at 8 × 10^4^ cells per well in a 24-well cell culture plate containing poly-D-lysine-coated round cover glasses (Menzel-Glaser, Avantor, Darmstadt, Germany) and performed SH-SY5Y cell differentiation. For the immunofluorescence staining, the cells were washed with 1× PBS and then fixed with cold 4% paraformaldehyde for 10 min. Following fixation, the cells were incubated with a blocking solution containing 1% BSA in PBST for 45 min. Next, the cells were incubated with the primary antibody, NRF2 (D1Z9C) XP^®^ Rabbit mAb, at a 1:300 for 18 h at 4 °C. After primary antibody incubation, the cells were treated with the secondary antibody, goat anti-rabbit IgG H&L (Alexa Fluor^®^ 488), at a 1:1000 dilution for 2 h in the dark at room temperature. Following three washes with PBS, the cells were incubated with TO-PRO-3 dye for nuclear staining, diluted 1:1000 in 1× PBS, for 30 min. Finally, the stained cells were rinsed with 1× PBS, and the slides were mounted with glycerin and protected from light exposure. The stained cells were visualized and recorded using an Inverted Confocal Laser Scanning Microscope FV1000 (Olympus, Tokyo, Japan). The positive signals with green fluorescence were then quantified using ImageJ software, version 1.52a (Java 1.8.0_112). The background signal was then subtracted, and the following formula was used to calculate the ratio of the average green fluorescence intensity between the nucleus and the cytoplasm to determine the average signal value.

$$\sum_{i=1}^{N}\frac{nucleus-bk}{\frac{cytoplasm-bk}{N}}$$

From the above formula, ‘nucleus’ is the mean green value measured for each individual nucleus, ‘cytoplasm’ is the mean green value measured for each cytoplasm, ‘bk’ is the mean of background signals, and N = 100.

### 4.10. Quantitative Real-Time PCR (RT-qPCR)

In order to obtain precise, quantitative data on the gene expression levels of antioxidant genes *SOD1*, *GST*, *GCLC*, *GPX3*, *HMOX1*, and *NQO1*, RT-qPCR was performed. In multiplate 96-well PCR plates (Bio Rad, Berkeley, CA, USA), the reaction mixtures were added along with ultrapure distilled water, the cDNA templates, forward and reverse primers, and iTaq Universal SYBR Green Supermix (2×). The primer sequences utilized in this study are as follows: SOD1 (FW: 5′-GATGACTTGGGCAAAGGTGG-3′, RV: 5′-TACACCACAAGCCAAACGACT-3′) [61], GST (FW: 5′-AGGTGACACTATAGAATAATACATGGCAAATGACTTAAA-3′, RV: 5′-GTACGACTCACTATAGGGATGATGTCTTCATTCCTTGAC-3′) [62], GCLC (FW: 5′-ACATCTACCACGCAGTCAAGG-3′, RV: 5′-CTCCAGAGGGTCGGATGGTT-3′), GPX (FW: 5′-GCAGAGCCGGGGACAAGAGAA-3′, RV: 5′-CTGCTCTTTCTCTCCATTGAC-3′) [63], HMOX1 (FW: 5′-GCTCAAAAAGATTGCCCAGA-3′, RV: 5′-GCTCTGGTCCTTGGTGTCAT-3′) [63], NQO1 (FW: 5′-AGGTGACACTATAGAATACTGCGAACTTTCAGTATCC-3′, RV: 5′-GTACGACTCACTATAGGGAGAAGGGTCCTTTGTCATAC-3′) [62], and GAPDH (FW: 5′-GAATGGGAAGCTGGTCATCAA-3′, RV: 5′-CCAGTAGACTCCACGACATACT-3′) [62]. The Bio-Rad CFX (Berkeley, CA, USA) was used to perform RT-qPCR under thermal cycling conditions of 95 °C for 10 s (denaturation), 65 °C for 10 s (annealing), and 72 °C for 30 s (extension). Following the completion of these steps, the procedure was repeated for a total of 39 cycles. Data were analyzed using the 2^−∆∆Ct^ method, with GAPDH serving as the normalization control. All reactions were carried out in triplicate.

### 4.11. Statistics Analysis

All results are presented as the mean ± SEM and were analyzed using IBM SPSS Statistics 26. For comparisons between two groups, a *t*-test was applied, while one-way ANOVA, followed by the Least Significant Difference (LSD) test, was used for comparisons involving three or more groups. A *p*-value of ≤0.05 was considered statistically significant.

## 5. Conclusions

This study shows the possible application of WPI in the treatment of oxidative stress in a simulated PD model. The dopaminergic-like neuron differentiation of SH-SY5Y cells was confirmed by the increased expression of TH protein and *DRD2* and *SLC18A1* genes. WPI showed strong antioxidant activity through the stimulation of Nrf2 nuclear accumulation and, consequently, the Nrf2 signaling pathway and genes encoding antioxidant enzymes, GPx and HO1. There was a substantial increase in the intracellular ROS levels on account of the increased expression of these genes, which shows that WPI is effective in combating oxidative stress. These findings indicate that WPI can be considered as a potential source of bioactive compounds aimed at the Keap1-Nrf2 pathway in order to protect neuronal cells from oxidative damage and death.

## Figures and Tables

**Table 1:** Amino acid composition of WPI.

Amino Acid Profiles	Concentration (mg/100 g)
Glutamic acid	17,457.40
Aspartic acid	10,920.43
Leucine	10,441.27
Lysine	8342.23
Threonine	7006.69
Isoleucine	5808.87
Proline	5747.21
Valine	5319.86
Alanine	4955.06
Serine	4510.00
Cystine	3323.42
Phenylalanine	3206.23
Tyrosine	2957.29
Methionine	2205.17
Arginine	2109.95
Histidine	1872.83
Glycine	1705.66
Tryptophan	1536.06
Hydroxylysine	574.38
Hydroxyproline	Not detected

## Data Availability

The original contributions presented in this study are included in the article. Further inquiries can be directed to the corresponding author.

## Abbreviations

The following abbreviations are used in this manuscript:

WPI	Whey protein isolate
MPP^+^	1-methyl-4-phenylpyridinium
Nrf2	Nuclear factor erythroid 2-related factor 2
Keap1	Kelch-like ECH-associated protein 1
